# New Appliances and Things Medical

**Published:** 1894-04-28

**Authors:** 


### New Appliances and Things Medical.

[All preparations, appliances, novelties, &c., of which a notice is desired, should be sent for the Editor, to care of The Manager, 428, Strand, London, W.C.]

### TEA.

The United Kingdom Tea Company, 21, Mincing Lane, London, E.C.

Several samples of tea have been forwarded to us by the above firm. We have examined and tasted three varieties, namely, Souchong Assam and Ceylon Pekoe (No. 7 on the company's list), Kaisow and Darjeeling (No. 4), Choicest Ceylon and Finest Darjeeling (No. 5). All of these teas we have found perfectly pure samples, good quality of leaf, and excellent flavour, but perhaps the most remarkable feature of the company's teas is the price. By dealing directly with the merchant, without the intervention of the middleman, the public is enabled to feel the benefit of the late reduction in the tea duty, and in buying teas from the above firm, who have got a first-class reputation to lose, the buyer may rest assured he will get what he pays for.

### FLORADOR FOOD.

(Florador Food Company, Washington Mills, Glasgow.)

This is a very excellent starchy preparation, containing a high percentage of gluten, or the albuminous constituent of flour. It may be used in those cases where arrowroot or cornflour are generally employed—namely, for making cakes and puddings. The manufacturers are willing to forward samples of their food free of charge to all who may care to try this preparation. Each packet of the food is provided with a number of receipts which will be new to many and acceptable to all, as dishes for breakfast, lunch, and dinner in useful variety are included in the list.

